# Exploring the stress of olympic postponement due to COVID-19 on elite/international and world-class parenting and pregnant runners

**DOI:** 10.3389/fspor.2023.1001127

**Published:** 2023-04-11

**Authors:** Francine E. Darroch, Sydney V. M. Smith, Madeleine D. Sheppard-Perkins, Audrey R. Giles, Dylan Wykes

**Affiliations:** ^1^ Department of Health Sciences, Faculty of Science, Carleton University, Ottawa, ON, Canada; ^2^ School of Human Kinetics, Faculty of Health Sciences, University of Ottawa, Ottawa, ON, Canada; ^3^ Mile2Marathon Coaching Inc., Ottawa, ON, Canada

**Keywords:** stress, athletics, COVID-19, parents, olympics

## Abstract

The primary objective of this community-based participatory research is to explore the impacts of COVID-19 and the delayed Tokyo 2020 Olympic Games on world-class and elite/international-class parenting and pregnant athletes. Participants in this study include 11 female and 10 male parenting and/or pregnant middle and distance runners. Combined, the participants have competed at 26 Olympic Games and 31 World Championships. Drawing on the general concepts of stressors and psychological resilience, we use thematic analysis to develop four themes to understand the stressors for world-class and elite/international-class parenting and pregnant athletes due to COVID-19 and the delayed Tokyo 2020 Olympic Games: (1) lack of childcare support, (2) family planning, and (3) needing to stay away from sources of COVID—including their children. Despite the stressors identified in the aforementioned themes, we identified a fourth theme: (4) participants demonstrated adaptability to stress in spite of—or due to—their athlete-parent identities.

## Introduction

The novel Coronavirus disease (COVID-19) outbreak has had significant and ongoing impacts on the global sport and exercise world, with international quarantine and lockdown requirements^1^ having prompted decisionmakers to re-imagine the landscape of recreational and professional sport (1–3). Safeguarding strategies have included the widespread cancellation or postponement of large-scale sporting competitions such as major marathons, professional sport leagues, the 2020 Summer Olympics, and other national and international games (4, 5). Beyond the evident economic consequences of these cancellations, the pandemic has also had notable social impacts, with professional sport being a mechanism for some forms of social cohesion, community development, and physical activity participation amongst participants and fans (6). For elite athletes, however, critical impacts include stressors related to social isolation; significant career interference; uncertainty in sporting qualification processes and professional contract renewal; limited or unconventional access to training facilities, teammates, and coaches; and changes in competitive schedules (7–10). The challenges of the COVID-19 context have reached even further for elite athletes who are pregnant and/or parenting as these individuals have uniquely faced a multitude of overlapping stressors related to the numerous commitments and responsibilities that come with both elite performance/training as well as parental duties. While researchers have explored the varied stressors of elite athletes more broadly, there is a paucity of research specifically focused on elite pregnant and/or parenting athletes in particular.

## Literature review

### Stress and stressors

The field of stress research is vast, and definitions of stress thus vary. The World Health Organization (11) defined work-related stress as “the reaction people may have when presented with demands and pressures that are not attached to their knowledge and abilities and which challenge their ability to cope” (para. 1). McEwen's (12) definition of stress included the idea that it can have physiological, psychological, and behavioural elements: stress is a “real or interpreted threat to physiological or psychological integrity of an individual which results in psychological and/or behavioural responses” (p. 508). Importantly, stress is a subjective experience, meaning that even if a number of individuals are exposed to the same stressor, responses will vary by individual (13). Social stressors are often identified as falling into three main categories: life events, chronic strains, and daily hassles (14, 15). Life events are described as changes that require adjustments within a short period of time (e.g., job loss, having a baby, or a global pandemic). Chronic strains are persistent and repetitive demands that necessitate adaptation over longer periods (e.g., constant work demands, family stress). Daily hassles are minor events and occurrences that require adjustments throughout one's day [e.g., arguments or inconveniences; (14)]. While these social stressors are conceptually distinct, they may work synergistically to impact individuals and have cumulative effects on their health.

### Stress and athletic environments

Elite athletes experience high levels of stress (16). Athletic environments are dynamic, and athletes' responses to changing stressors contribute to their adaptability and success in their respective sport. In a recent systematic review, Singh and Conroy (17) examined stress-related injury vulnerability in athletic and occupational contexts. They found a positive association between stress and occurrence of injury. Indeed, as noted above, stress responses can present as psychological, physiological, or both—which may in turn increase risk of injury (17). Soligard et al. (18) conducted a consensus statement on load in sport and risk of injury on behalf of the International Olympic Committee and stated that the “mechanism by which psychological stress responses increase injury risk is through attentional and somatic changes such as increased distractibility and peripheral narrowing, as well as muscle tension, fatigue and reduced timing/coordination” (p. 1035). The authors further examined “load” in sport and risk of injury and noted that in addition to management of training and competition loads, considerations of non-sport stressors, such as negative life-event stress and daily hassles, need to be taken into account when considering stress on athletes. In addition to this, they noted that other psychological variables such as personality variables, stress susceptibility, and type A behaviours can impact risk of injury. The researchers discussed non-sport load stressors but did not mention the impact of stress due to pregnancy and/or parenting on risk of injury for athletes. Indeed, the first recommendations in the general guidelines for illness prevention in athletes is to “minimise contact with infected people, young children, animals and contagious objects” (18), which is challenging if you are a parent.

### Stress of olympic postponement on elite athletes

Elite athletes have faced unique challenges in the face of the COVID-19 pandemic and the postponement of the Tokyo 2020 Olympic Games. The stressors brought on by the pandemic shed light on how proficient high-performance athletes can be at mitigating the potential effects of anxiety on performance (7, 19, 20). For some athletes, the delayed Olympics meant an extra year for additional training and preparation for the Games or to attend to injuries that may have threatened their ability to compete (21, 22). But for others, the postponement marked a “significant career disruption” leading to potential “loss of identity, motivation and meaning” (23). Rogers and Werthner (24) highlighted the importance of individual context for elite athletes' experiences preparing for the Tokyo Olympics, highlighting social support, being more than an athlete, and “learning to reflect on one's life” (p. 1).

Alongside the possibility of having to navigate the process of loss, high-performance athletes have also had to manage their usually meticulously calculated, thought-out, and structured training plans being in turmoil. Athletes thrive and progress according to very strict and consistent short- and long-term planning (8), which was utterly derailed by the prevailing state of uncertainty before, during, and after the official announcement of the postponement of the Olympics (21). Specifically, researchers have highlighted the multiple strategies athletes engaged with to maintain fitness during lockdowns such as body-weight-based exercises, practicing technical skills, and cardiorespiratory training—although these were completed at levels lower than pre-pandemic (4, 9, 10). The COVID-19 lockdown negatively impacted training for athletes including the inability to access facilitates, coaches, and other services required to maintain progression of fitness, which impacted mental health (4, 9, 10). High-performance athletes will typically “think [and therefore plan] in Olympic years” (25). With the entire four-year Olympic cycle being shifted, there has been growing concern for the profound levels of psychological distress that athletes may have experienced beyond feelings of anguish that were directly related to challenges to their athletic identity and career in sport (26).

A major long-term repercussion for some athletes may have included having to postpone or adjust other life plans, including starting or growing a family (23, 26).

### Family planning and management stress on elite athletes

Less recognized considerations of COVID-19-related global sporting disruptions include the logistics and management of family planning for elite athletes. For this specific population, factors such as peak performing years, relative fertility age, and timing of major events such as the Olympics and national championships critically inform decisions surrounding family planning and pregnancy (27, 28). Researchers have documented the stressors parenting and pregnant elite athletes experience (including related to family planning), even outside of the COVID-19 context (27, 29–33). Indeed, the effects of COVID-19 on family planning are far-reaching, with a specific set of issues influencing attitudes, perceptions, and behaviours surrounding pregnancy intentions. These considerations range in nature and include but are not limited to the uncertain physiological and immunological effects of COVID-19 on maternal, fetal, and/or newborn contraction of the virus (34–36); the overburdening public health systems (37); fewer birth supports during labour and delivery (38); and lack of postpartum supports (39, 40).

To ensure that they are in peak fitness when it matters most, the very element of uncertainty in parenthood may drive an elite athlete's family planning to specifically revolve around key competitions or major events—such as the Olympic Games. Athletics Canada's High Performance Director Simon Nathan touched on the major sacrifices that elite athletes make in pursuit of qualifying for the Games and how this sacrifice is further influenced by COVID-19: “they park all sorts of things, they park their life, having families, their education, their careers to make this go. And because the Games were postponed, they had to stretch that for another year” (41). There is a need for further research to understand how elite athletes handle stress surrounding family planning and parenting, particularly within the pandemic context.

### Parenting stress and COVID

There is growing evidence of the various ways that the COVID-19 pandemic—and associated social restrictions, quarantine requirements, lockdowns, and general health concerns—has contributed to substantial increases in stress levels for individuals who are pregnant (42, 43) and/or parenting (44–46). While scholars have noted that the magnitude of such increases in parenting stress varies between families of differing socioeconomic status (47), increased stress has nonetheless been felt by all parents in one way or another, including those of young children (48–50), adolescents (51), and foster children (52). The unprecedented situation of the pandemic has presented as a nearly perfect storm of events that have contributed to rising stress levels for parents. Between financial instability (46, 48), school closures (44, 45, 50), and major changes in children's daily routines (44), parents have had no choice but to fall into a “collision of roles” (53) head-on—from attending to parent-related responsibilities to those of an employee to partner/spouse. The increases in parental stress levels as a result of the pandemic are of serious concern, especially given their close relation to increases in symptoms of anxiety and depression (45, 54, 55).

### Psychological resilience

Fletcher and Sakar (56) defined psychological resilience as “the role of mental processes and behaviour in promoting personal assets and protecting an individual from the potential negative effect of stressors” (p. 675). These researchers developed a model of sport resilience based on research findings with Olympic Champions, which considers positive personality, confidence, focus, motivation, and perceived social support as crucial elements to developing resilience (56)—and recognizing the dynamic nature of the process (57). As such, part of this process identifies that an individual's ability to adapt to the challenges and circumstances they encounter is a capacity that develops over time with specific considerations of context. Drawing on the construct of psychological resilience, Rogers and Werthner (24) aimed to understand athletes' experiences preparing for the Tokyo Olympics and identified the ways in which participants in their study develop resilience. Their findings indicated the importance of life context, the crucial nature of social support, the significance of being more than an athlete, and the importance of learning to reflect on one's life as an athlete and beyond. Given the evidence of substantial increases in stress for parents more generally and unique stressors of the Olympic delay on athletes in particular, drawing on Fletcher and Sakar's (56) psychological resilience concept, in this paper we provide a novel examination of stress specific to elite/international to world-class athlete-parents in the context of a global pandemic.

## Methodology/methods

We obtained ethics approval for this study from Carleton University Research Ethics Board and University of Ottawa Research Ethics Board. The results presented in this article are part of a larger community-based participatory research (CBPR) project with pregnant and parenting elite/international to world-class runners. CBPR is an approach to research whereby community members play an active role in all aspects of the research process including determining research priorities, developing research questions, and supporting the collection as well as the interpretation of data (58). A community advisory board (CAB) comprised of five elite/international to world-class parenting athletes (two men and three women) and three academics guided all aspects of this research. CAB members hailed from Canada, USA, and Ireland. The CAB met formally to determine priority research areas at the initiation of this project. The CAB members also contributed to and approved the interview guides, supported recruitment, and provided feedback and insights on the analysis and findings.

As the focus of this project was to explore the stressors for elite/international to world-class parenting and pregnant athletes due to COVID-19 and the delayed Tokyo 2020 Olympic Games, the inclusion criteria for this study were the following: English speaking, currently pregnant and/or parenting, and meet the criteria for elite/international to world-class middle- to long-distance runner as developed by McKay et al. (59). Based on these categorizations of our 21 participants (see Table 1), 12 are Tier Four (Elite/International level, which means they are competing at the international level, top 4–300 in world rankings, within approximately 7% of world-record/world-leading performance, NCAA Division 1 athletes); and 9 participants are Tier Five (World-Class, which means they are Olympic and/or World Championship medalists, world record holders, and/or top 3–10 at an Olympics or World Championships). We purposively recruited (60) participants through the CAB's personal networks. Further to this, snowball sampling (61) occurred. The first and second authors conducted 21 semi-structured interviews (62) with pregnant and/or parenting individuals, including 10 males and 11 females, hailing from four countries, between March 2021 and January 2022 *via* Zoom software or on the telephone. The interviews ranged in length between 45 and 95 min. We asked specific questions related to the impacts of COVID-19 and the delayed 2020 Tokyo Olympic Games on running career plans, parenting, family planning, and stress (see supplementary file 1). We digitally recorded each interview, transcribed them verbatim, and then checked them for accuracy. We sent all participants their transcripts for quality checking; none of the athletes requested changes to the transcripts. All participants received a $25 gift card to thank them for their time.

**Table 1:** Participant demographics.

ID	Category (at the time of interview)	Participant Pseudonym	Categorization
001	Pregnant	Mallory	World Class
002	Father	Darren	Elite/International level
003	Mother	Mary	World Class
004	Father	Roger	Elite/International level
005	Mother	Sally	Elite/International level
006	Mother	Michelle	Elite/International level
007	Mother	Natalie	Elite/International level
008	Father	Ricardo	Elite/International level
009	Father	Charles	Elite/International level
010	Mother	Monica	World Class
011	Father	Martin	Elite/International level
012	Mother	Katherine	Elite/International level
013	Father	Noah	World Class
014	Mother	Lydia	World Class
015	Father	Elias	World Class
016	Mother	Sophia	Elite/International level
017	Father	Adrian	World Class
018	Mother	Hannah	World Class
019	Father	Caleb	World Class
020	Expecting Father	Cory	Elite/International level
021	Pregnant	Renee	Elite/International level

## Analysis

As social constructionists who recognize the role that one's positionality may play in one's research, we note that this research was spurred by the authors' knowledge of and experience in high-performance sport. The first author is a former elite/international runner and is married to a 20XX (anonymized) Olympic runners. They have young children and direct experience with issues discussed in this manuscript. Author two is a national-level runner, author three has been involved in competitive sports, author four was a national-class varsity runner, and the last author is an advisory board member, a father, and an Olympian.

We used Braun and Clarke's (63) six-step approach to thematic analysis. Thematic analysis allows researchers to describe, organize, and interpret qualitative data to understand descriptions of participant experiences through constructing common themes and categories (63). Through this systematic approach, we developed, analysed, and reported patterns of meaning within the data (63). We used Nvivo qualitative software to code the data. All authors reviewed the codes (family planning, challenges, finances, resiliency, adaptation, children as priority, unpredictability, supports), which we then used to generate four themes to understand the stressors for world-class and elite/international class parenting and pregnant athletes due to COVID-19 and the delayed Tokyo 2020 Olympic Games.

## Results

We developed four themes to understand the stressors for elite/international to world-class parenting and pregnant athletes due to COVID-19 and the delayed Tokyo 2020 Olympic Games: (1) lack of childcare support, (2) family planning, and (3) needing to stay away from sources of COVID—including their children. Despite the stressors identified in the aforementioned themes, we identified a fourth theme: (4) participants demonstrated adaptability to stress in spite of—or due to—their athlete-parent identities.

### Lack of childcare support

COVID-19 affected the participants' training and parenting in unprecedented ways. Many of the athlete-parents took on the additional roles of childcare provider, educator, and playmate for their child(ren), which limited their time for training. The parent-athletes noted that their typical training schedules were challenged if they did not have partner or childcare support. One mother, Monica, described her situation whereby she lived far from extended family:

We didn't have daycare … so my runs were done on a treadmill when she [child] was napping because he [husband] was still working. He was [an] essential [worker] … On the days he was off … it was very normal. I could do what I needed to do, but, um, it was very difficult during COVID to get done what I needed to get done, because I was just exhausted from parenting all the time … By the time my time came, my two hours to do what I needed to do to work out … I was done, like mentally I was drained, physically I was tired, and … it was really hard.

Similarly, Sally noted, “it was super stressful, like that, you know, balancing [children's online] school and [my own] training.” As these participants indicated, the lack of supports during this time were particularly challenging as they navigated the COVID landscape of school and daycare closures with limited childcare support. One of the fathers, Darren, had a similar experience, noting that he could no longer prioritize his training with the uncertainty of the Olympic Games: “With the Olympics not being like the number one focus for me … it's harder to say, okay, let's rejuggle everything, and just focus on this [training].”

Participants also discussed the financial stress related to childcare. Although not speaking to his own situation, Noah noted the additional financial stress on some athletes due to their children being out of school. He stated, “the extra income [is] needed to pay for babysitters, nannies, daycare, whatever it is. And as an athlete, there already is a lack of funding.” Unlike some other professional sports, middle- to long-distance running is not a lucrative endeavour, and many athletes did not have the means to hire childcare providers when schools and daycare facilities were shut down. Numerous athletes noted that the resulting stress was overwhelming. Those who had the means to do so opted to hire additional in-home childcare supports. As Hannah noted, “We did the best we could, but it was … stressful with scheduling. We finally ended up getting a nanny. So [that] added cost, like I think we spent like 30 grand on childcare in one year.”

Two parent-athletes argued that the overwhelming stress of COVID, the Olympic postponement, and parenting led to injuries. One dad, Martin, noted, “it's probably not surprising that I actually got injured for quite a big period of 2020. I think just, just stress and everything that was going on took its toll and yeah, so it was unfortunate.” Similarly, Mary noted an injury in 2020 that was stress related: “I actually attribute that [injury] largely to COVID stress.” These parents demonstrate how the increased levels of stress, anxiety, emotional exhaustion of parenting, and the additional childcare stressors in conjunction with training at an elite/international to world-class level during the current pandemic sometimes manifested in physical injuries.

### Stress of family planning

The postponement of the Tokyo 2020 Olympic Games affected family planning for some participants. For many participants, pregnancy considerations pertain to timing for personal life circumstances, relative fertility age, and major championship cycles—such as the Olympics. Given this, many athletes chose to delay pregnancy to attempt to qualify for and/or compete at the Olympics. Monica explained the direct impact the delayed Tokyo 2020 Olympics had on her family planning: “We did have to put things off by a year and, you know, at some point we would like to expand our family, and we knew that the Olympics was the next thing on my radar.” This mother had planned her second pregnancy to be post-Olympic Trials/Olympics, but when the Olympics were delayed, she was forced to choose between the Trials and pregnancy. Similarly, Sally acknowledged,

I know for a lot of women who, you know, haven't started their families yet or plan things around the Olympic cycle, like I fully can acknowledge and see how that [delay] completely changed a lot of women's plans. … We're done having babies now. But if we were in the position where I was planning on having another one, uh, that would have really screwed things up because it probably would have been planned for next year [2021]. And everything got shifted back a bit. There's no break year [first year of a four-year Olympic cycle] there for us now.

The one-year delay of the Olympic Games caused significant distress for some athletes more than others. As Mallory noted,

I mean ideally, like, I would have tried to have [a baby]. I mean, I'm an athlete kind of towards the end of my career, so it feels a little more urgent. Like maybe some of the young athletes are okay to wait another year or two … [but] you don't want to wait too long.

This participant affirmed the stresses, particularly for female athletes, in delaying pregnancy with consideration of optimal years of fertility.

While father-athletes do not have to deal with the physical repercussions of bearing a child, there are significant considerations of timing, nonetheless. Charles noted,

When the Olympics were going to be in 2020, we were thinking about trying for a family after that so that we could have a bit of downtime and plan it … we were trying to look to have a child in a, in a much more quieter time of the running season and create some kind of stability in a very unstable environment [COVID-19] with running … especially me being older, we didn't want to wait too much longer. When we found out we were pregnant at that time, we didn’t know when the [Olympic] Trials were, we didn't know if the Olympics were going to go ahead. So, when we saw the date of his due date, and we started seeing that the Trials were going to be the week of his arrival, it suddenly dawned on us that the one thing we were trying to avoid of everything happening at once was … happening now.

While there were numerous challenges related to family planning, the athletes also discussed the stress of navigating parenthood amid an Olympic year in a pandemic context.

### Staying away from COVID and children

The participants in our study had the added stress of increased risk of exposure to COVID-19 from their children in school and/or childcare settings, which had the ability to derail their competition plans. Katherine noted that there were multiple exposures to COVID-19 in her children's school. She explained,

My daughter had a positive case in her class right before I was to go to [city] to race the marathon. … That increases the stress level … We want to keep our distance, especially from our kids that can carry a lot of germs. Basically, for the first couple of days, until she tested negative, I had her wear a mask, and I kept my distance from her because even though I'm vaccinated, you know, I can still get it. And if she had it and I was a close contact, I wouldn’t have been able to go. And that would have been devastating for all of that training.

Parent-athletes dealing with travel to competition face some unique challenges, including being unable to travel with their children. Mary shared,

I'm not used to being away from my kids for one thing. And I like being around them. … [If] the world was the way it had been [pre-pandemic], I would have absolutely wanted them to be around [at competition] because I’m just used to it. Right … the second thing that may be difficult is if, especially if things get stressful at home and I'm not there to help, and I hear about it, like I'd rather just not hear about how difficult the days have been without me around. Right? Just lie to me. Just say, “it's going really well.”

Breastfeeding mothers faced specific stressors related to a lack of evidence and advice for postpartum mothers. As Sally explained,

I was nursing at the time [early in the pandemic], and I just felt uncomfortable and could not get any clear advice on whether or not it … was okay for me to fly to a race in [US state], and then fly back and nurse right away. Like, they just didn't know if I needed to self-quarantine. And so just the idea of like, the possibility of having to quarantine away from my kids, but being at home, like the idea of that was too much. And I was like, there's no way I could even risk that. Because you know I did have a doctor, like I reached out to my doctor and pediatrician for advice. And it was just, they were like, yeah, you should, it's better safe than sorry, especially if you're nursing like … to go two weeks quarantining and not nursing, and they didn't know if it [COVID-19] was in breast milk, or if it was contact or like how it could spread to babies. And I just didn't feel like it was worth the risk. And so that made me really nervous, like the fact that I was nursing and thinking about travel.

Most parents in this study spoke about the stress of additional hassles associated with quarantines. Darren discussed the complexity of finding a race to meet his country's Olympic qualification criteria and the need to travel:

To travel outside of Canada and then to come back and have to quarantine for two weeks would be really difficult for our family. So basically, we would have to keep the kids home as well for those two weeks and keep them out of school … which would be tough for everyone. So, it's like, it's definitely something that would have to be considered more … I would feel like I would have to be 100% confident in picking a race and going to a race that, that I'm gonna, you know, run my best race and that it's not going to be some wasted opportunity and then I'm stuck having, you know, made the situation for my family more difficult after the fact. It'd be a big burden for them.

As this father explained, his decision making was not focused purely on choosing the best race to meet the Olympic standard; rather, he considered the implications of travel and quarantine times on him and his family. Roger, who we interviewed while he was in quarantine after a major race and Olympic qualifying attempt, described his mid-race decision-making process about making another Olympic qualifying attempt and the impact it would have on his family:

In the race, when I knew I wasn't going to get the Olympic standard, part of me really wanted to drop out … I had these thoughts of dropping out, and part of it was if I dropped out at around 30 K, I could rebound and then maybe do another race … In normal circumstances, you know, I would've cut my losses, easily recovered, you know, relatively easily recovered after a 30 K effort vs. 42 K and, and go there [another race]. But because I knew I was gonna have to quarantine for two weeks, that's going to limit training and then put my family through this whole ordeal, again, five weeks later for an uncertain result. … I just wasn't going to do it.

This father relayed the specific considerations of competition-related stressors, the strain on his family, and the backdrop of the COVID-19 travel context, which all informed his decision to prioritize his family over another Olympic qualifying attempt.

### Stress and adaptability as athlete-parents

The participants in our study demonstrated remarkable adaptability in the face of significant stressors. Many of them attributed this adaptability to being both parents and athletes. Sophia noted that being adaptable is essential to being a professional athlete and parent:

It's that old saying, like, “life happens when you’re busy making other plans.” So, I kinda just had to adapt and, luckily being a professional athlete and a mom, those are two of your number one, uh, requirements for job description is being adaptable.

Caleb noted that parent-athletes are likely faster at adapting to unexpected stressors than their non-parenting peers:

I think probably being a parent, you’re just like, okay, well let's adapt and, and be quicker at adapting. Um, I think athletes … who hadn’t had to adapt before, i.e., bringing a human into the world, they were just like, “Oh my gosh, this is terrible! What are we gonna do? I can’t handle this.” Um, but as a parent, you're kind of just like, alright, well, you know, let's get our ducks in a row, make sure everything's taken care of and you just, you know … my wife and I, our, our motto is just, just get on with it.

Another father, Elias, noted that he did not find there to be too much of a difference between COVID-19-related quarantines and the usual demands of being a professional athlete:

Yeah, it's unpredictable, isn’t it, being an athlete? So, I guess we’re used to being in such a volatile position—I just told myself I just need to be ready. So, if I was ready, if the Olympics happen that year, then I just made sure that I was ready, and I just wanted, I hoped that my competitors weren't ready. So, I made sure I was. Yeah, it was, it was quite intense. Um, so there were lots of quarantines. However … I guess being an athlete, you're almost in quarantine permanently anyway, because you train once or twice a day. And then the rest of it is learning how to do nothing and just sort of chilling out. So overall, um, life didn't change too much.

Charles revealed that having the Olympics pushed back a year gave him time to adapt to major life changes, including having a child, which actually alleviated some of his stress and allowed him to enter 2021 feeling refreshed:

I needed an extra year to prepare for the Olympics … it gave us a chance to just pause life for a bit and let us catch up. We're always like, oh, we go from one championship to another where, you know, it's just relentless day after day. And it kind of gave us a moment, right when we were getting to the point of big life changes of moving house, having a child, changing career, there were so many big things about to happen. And it just gave us a second just to press the pause button and do it. And I think that's [a] big part of the reason why we, we went into 2021 fresh.

Despite the multifaceted challenges that elite/international to world-class parenting and pregnant athletes faced in dealing with athletic careers tied with parenting in a pandemic context, and delayed Olympic Games, they demonstrated adaptability and resiliency.

## Discussion

The ever-shifting demands of being an elite/international or world-class pregnant or parenting runner in the context of a global pandemic led to physical, mental, and emotional stressors that were experienced by participants in our study in a myriad of ways. The stress of COVID-19 and the resulting disruptions and impacts on athletes more broadly have been well-cited in academic literature (26, 64). The stress of life events, as described by Carr and Umberson (14), require adjustments within a short period of time, as experienced by our participants in the initial stages of the pandemic. Moreover, the literature has made it clear that the stress of pandemic restrictions has disproportionately burdened parents of young children, particularly mothers (42, 44, 46, 50, 54, 65). The “event” of a global pandemic has led to chronic strains, the persistent and recurring demands that have required constant adaptation across the (ongoing) pandemic, and an increase in daily hassles. Indeed, the participants in our study were no strangers to the multifaceted stress of the pandemic as they experienced varying levels (albeit equally strict and severe) of restrictions and public health guidelines across the USA, UK, and Canada. Importantly, and specific to parenting and pregnant athletes, the stressors identified by our participants due to COVID-19 and the delayed Tokyo 2020 Olympic Games included a lack of childcare support, family planning, and needing to stay away from sources of COVID—including their children. Despite these identified stressors, the elite/international or world-class parenting and pregnant athletes who participated in our study demonstrated adaptability and resiliency. Below, we discuss our results.

### Childcare supports

In our study, athlete-parents' ability to cope with stress was contingent on social and financial supports. In line with Purcell et al.'s (66) findings, the presence of adequate social support (including partners) can significantly reduce the manifestation of mental health challenges or psychological distress in the lives of elite athletes. While this point speaks to all elite athletes, it certainly carries weight for parenting athletes in particular, as exemplified by the elite athletes who have expressed their reliance on their personal support networks to assist them throughout their athletic and parenting pursuits [e.g., (27, 29, 67–69)]. Our findings advance this research by including the importance of supports in facing the unique challenges of the COVID-19 global pandemic and unprecedented delay of the Tokyo 2020 Olympics. Moreover, financial supports have also been cited as crucial for parenting athletes to continue their athletic careers (27). Our research highlights the increased importance of financial supports in the COVID context, with increased need for childcare supports due to daycare and school closures.

Elite athletes spend a great deal of time engaged in planning to cope with stressors that can detract from their performances (70). The inability to plan was amplified in a COVID-19 context wherein athletes face so many unknowns, particularly parents who were dealing with shifting restrictions and school/daycare closures, which increased chronic strains. In a recent systematic review, Singh and Conroy (17) examined stress-related injury vulnerability in athletic and occupational contexts. They found a positive association between stress and occurrence of injury. Several athletes in our study noted that they believed that the stress of balancing elite-level training with the increased demands for childcare and general stress led to injuries. Stress responses can present as psychological, physiological, or both—which may, in turn, increase risk of injury (17). Further research should examine the specific stressors and physiological impacts for this population.

### Family planning

Athletes' family planning and management was complicated by the impacts of COVID-19 and the delayed Tokyo 2020 Olympic Games. Many elite athletes plan their pregnancies around Olympic and World Championship cycles (27, 71). Participants spoke to the specific considerations and stress of delaying plans for pregnancy. Some female athletes in our study identified family planning as a biological concern with considerations of their age. In addition to planning around peak fertility years, female athletes must consider physiological, musculoskeletal, and psychological adaptations post pregnancy (71). Clearly, female athletes face distinct biological challenges compared with their male counterparts. However, our findings indicate some male athletes also consider family planning based on personal life timing and Olympic cycles. While researchers have examined the parenting experiences of mothers, who have been burdened with parenting pressures in the pandemic and have experienced greater stress than fathers (45, 46), as well as the elite/international to world-class (non-COVID) mothering context (29), our findings advance research in these areas to examine parenting experiences of elite/international or world-class athlete mothers *and* fathers.

### Staying away from sources of COVID-19

After the International Olympic Committee (IOC) announced that the Tokyo 2020 Olympics would incur a one-year postponement, moving forward with the Tokyo Olympics required the introduction of pandemic-related restrictions, including the exclusion of fans and family members from the event. The decision to ban spectators presented an important set of challenges for Olympians and their families. Breastfeeding athletes were among the most heavily impacted by this regulation, catalyzing major pushback from nursing mothers competing in Tokyo. Kim Gaucher, a member of the Canadian women's basketball team, spearheaded this protest, and she was quickly joined by cries of frustration from other Olympian parents and allies. In response to these calls for more inclusive policy, the IOC issued a statement confirming that, “when necessary, nursing children will be able to accompany athletes to Japan” (72). This issue drew major media attention to the importance of considering family planning and family care-related commitments for elite athletes, particularly in a COVID-19 context. While none of the athletes in our study who competed at the Olympics were still breastfeeding, many did note that in a non-COVID context they would have preferred to have their children with them at the Olympic Games. This particular situation highlighted the additional stress and considerations parent-athletes face. Further, the vast majority of participants noted the uncertainties related to races for qualification and travel limitations/restrictions, including the Olympic Games, and how they were further compounded by considerations of their families.

### Resilience

While the many challenges of COVID were certainly noted by all of the athletes in this study, there were also numerous occasions where many of them expressed noticeably positive outlooks on the unprecedented circumstance of the pandemic. For instance, although some athletes felt the delays negatively affected their chances to qualify for Olympic Trials or Tokyo 2020 Olympic Games, many felt that the delay enabled them to be in peak performance with additional time to recover from pregnancy or unrelated injuries. Other researchers have noted similar findings (21). The findings in our study align with recent research on non-parent elite athletes and the psychological stress of postponed Olympics and need for supports (7, 73, 74), but they importantly highlight the resiliency and adaptability of athlete-parents. Many participants spoke about the unpredictable nature of both athletics and parenting and the requirement to face head-on the challenges that come with this unpredictability—as such, the parent-athletes in this study adapted and “got on with it.” Importantly, when confronted with the unique intersection where the challenges of COVID coincide with the challenges of balancing parenthood with elite athletic performance, these parent-athletes demonstrated positivity, confidence, focus, and motivation—aspects that are all in line with Fletcher and Sakar's (56) crucial elements for developing resilience. Elite/international to world-class athletes are used to meeting the demands of competing in high-pressure environments; these coping strategies adapted from years as elite athletes may have helped parent-athletes cope with the multiple stressors they uniquely faced throughout the pandemic. Further, findings from Rogers and Werthner's (24) research noted “the importance of being more than an athlete” (p. 1) as a strategy to cope with adversity; thus, our research highlights that the identity of parent may protect athletes from some of the negative effect of stressors.

The study's limitations may provide direction for future research. The experiences of middle- to long-distance runners, who require very little equipment and can train on their own, may be quite different from athletes in other disciplines who compete in team sports, paralympic sports, and require access to specialized sporting facilities and equipment (10). Future research should examine the experiences of athlete-parents who play team sports or require sporting facilities and equipment and the role of social supports from teammates. Our findings were also limited to the exploration of the experiences of athletes who were located within the USA, UK, and Canada throughout the pandemic. As the severity of restrictions varied between different areas across the world, these findings may thus not be representative of the situations of other parenting and pregnant athletes outside of these three countries. Additionally, a direct examination into the degree to which athletes have access to various forms of support (e.g., corporate sponsorship, athletic governing body financial support, family supports, psychological supports) would be beneficial. Such future work could identify and address any gaps in athlete-parents' access to these supports, which may be critical in reducing this population's stress.

## Conclusion

Despite the varied lived experiences of participants and wide variety of COVID-related restrictions (e.g., lockdowns) based on geographic location, there were similarities and important considerations that contributed to the unique stressors experienced by elite/international to world-class parent-athletes. Participants highlighted stressors related to a lack of childcare support, family planning, and needing to stay away from sources of COVID—including their children. These findings demonstrate the unique experiences and support gaps for elite/international to world-class pregnant and parenting athletes and may assist sport governing bodies and sponsors in developing policies (e.g., policies specifically put in pace to support athlete-parents, such as introducing childcare) and changing the culture of athletics—which is currently not conducive to having children—to reduce stressors and provide adequate psychological supports to promote resilience, particularly in the context of an ongoing pandemic.

## Data Availability

The datasets presented in this article are not readily available; due to the nature of this research, participants did not agree for their data to be shared publically. Requests to access the datasets should be directed to francine.darroch@carleton.ca.

## Footnotes

^1^Many countries imposed different restrictions throughout the varying stages of the COVID-19 pandemic. Quarantine and self-isolation protocols were typically required for individuals who had either tested positive for COVID-19, were experiencing symptoms related to COVID-19, and/or had recently travelled and were thus assumed to have been exposed to and at a higher risk of becoming infected with COVID-19. Quarantine and self-isolation requirements involved the strict separation of an individual who was sick and/or exposed to COVID-19 from others who were not sick nor exposed. Lockdowns during the COVID-19 period involved stringent restrictions geared towards an entire community (e.g., closure of public spaces), with an overarching goal of preventing the spread of COVID-19 by limiting in-person travel and social interactions. The specific durations of time for quarantines and lockdowns varied depending on both the stage of the pandemic as well as between countries, provinces, states, and municipalities.
