# Supplementary material for: Exploring the stress of olympic postponement due to COVID-19 on elite/international and world-class parenting and pregnant runners
Source: Front Sports Act Living. 2023 Apr 11;5:1001127. doi: 10.3389/fspor.2023.1001127 (PMC10127675; doi:10.3389/fspor.2023.1001127)
Supplement: Supplementary file 1 [file Table1.docx]

**Semi-Structured Interview Guide:
*Questions Related to COVID-19***

Thank you for taking the time to participate in our study. The questions in this interview are closely related to issues that have been identified in the media including the impact of COVID-19 and delayed Olympics on life and running career plans as well as the additional stress of training in a COVID environment as parents. The overarching aim of this research is to understand the experiences of elite pregnant/parenting athletes. By gaining this understanding, the results of this study will have the capacity to inform and strengthen development within the sports industry to increase the level of support surrounding pregnancy, postpartum, and parenting elite athletes like yourself.

| **Demographic Information** |
| --- |
| We want to start off with a few questions relating to demographics.   - Firstly, what is your date of birth? |
| In what country do you currently reside?   - Is this the same country that you represent in international track competition? - What is your primary racing event? |
| How many children do you have?   - How old is/are your child(ren)? |
| Are you currently a professional full-time or part-time athlete?   - Are you currently employed in any capacity outside of athletics? |

| **COVID-19 Specific** |
| --- |
| Can you tell me about your current competition/training/work/parenting situation?   - Does this differ from your pre-COVID situation? |
| What has the impact of COVID-19 and the delayed Olympics had on your family and running career plans?   - How has this specifically impacted your competition and race plans? |
| How has additional stress of training in a COVID environment impacted your parenting and training? |
| What information has your athletic governing body relayed about quarantines pre/post Olympics?   - How will these rules/regulations impact your family life? |

Thank you again for taking the time to participate in our research study. We really appreciate your willingness to meet with us today and to provide insight as we hope that this will greatly contribute to enhancing parent-athlete experiences in elite running.
